# Supplementary material for: The influence of ecological infrastructures adjacent to crops on their carabid assemblages in intensive agroecosystems
Source: PeerJ. 2020 Jan 10;8:e8094. doi: 10.7717/peerj.8094 (PMC6956773; doi:10.7717/peerj.8094)
Supplement: Table S1 [file peerj-08-8094-s001.doc]

|  |  | Within 50 m around the crops | | | | | | Within 500 m around the crops | | | | | |
| --- | --- | --- | --- | --- | --- | --- | --- | --- | --- | --- | --- | --- | --- |
| Site | Association | % Crops | %  Meadows  ^(1)^ | %  Forests  ^(2)^ | %  Gardens | %  Ruderal vegetation | % Buildings | % Crops | % Meadows  ^(1)^ | % Forests  ^(2)^ | % Gardens | %  Ruderal vegetation | % Buildings |
| 1 | H | 86.32 | 13.60 | - | 0.07 | - | 0.01 | 78.71 | 16.70 | 0.60 | 3.64 | - | 0.35 |
|  | G | 100 | - | - | - | - | - | 80.58 | 14.55 | 2.44 | 2.29 | 0.04 | 0.09 |
|  | H-A | 85.72 | 13.51 | 0.77 | - | - | - | 90.56 | 8.09 | 0.73 | 0.55 | - | 0.07 |
|  | G-A | 100 | - | - | - | - | - | 94.73 | - | 4.71 | - | 0.56 | - |
| 2 | H | 99.39 | - | - | - | 0.61 | - | 89.83 | 7.45 | 0.48 | 1.68 | 0.40 | 0.17 |
|  | G | 96.93 | 3.07 | - | - | - | - | 88.40 | 8.00 | 0.21 | 3.10 | - | 0.29 |
|  | H-A | 88.55 | 8.85 | 0.72 | 1.84 | - | 0.04 | 79.14 | 11.46 | 2.09 | 5.79 | 0.85 | 0.67 |
|  | G-A | 99.03 | - | 0.34 | - | - | 0.63 | 78.12 | 9.67 | 5.19 | 6.13 | 0.37 | 0.52 |
| 3 | H | 100 | - | - | - | - | - | 93.55 | 5.95 | - | 0.41 | 0.02 | 0.07 |
|  | G | 95.24 | 4.76 | - | - | - | - | 95.27 | 3.88 | 0.40 | 0.27 | 0.12 | 0.06 |
|  | H-A | 89.27 | 10.73 | - | - | - | - | 92.26 | 6.59 | 0.21 | 0.71 | 0.12 | 0.10 |
|  | G-A | 76.17 | 23.00 | - | 0.83 | - | - | 89.99 | 9.25 | - | 0.59 | 0.04 | 0.12 |

Note : ^(1)^ Meadows encompass permanent and non-permanent grasslands; ^(2)^ Forests encompass: conifer cover, broad-leaved trees cover, mixed cover of broad-leaved trees and conifers, broad-leaved trees cover with and without undergrowth, heath and conifer, heath and broad-leaved trees.
