# Supplementary material for: The influence of ecological infrastructures adjacent to crops on their carabid assemblages in intensive agroecosystems
Source: PeerJ. 2020 Jan 10;8:e8094. doi: 10.7717/peerj.8094 (PMC6956773; doi:10.7717/peerj.8094)
Supplement: Table S2 — Functional traits are from the databases of Ribera et al. (1999) and Dufrêne (1992). [file peerj-08-8094-s002.doc]

| **Carabid species** | **Abbreviation** | **Mean size (mm)** | **Size**  **class** | **Diet** | **Wing morphology** | **Breeding period** |
| --- | --- | --- | --- | --- | --- | --- |
| *Agonum muelleri,*  (Herbst, 1784) | Agomue | 8 | 2 | Omnivorous | Macropterous | Spring |
| *Amara aenea,*  (De Geer, 1774) | Amaaen | 8 | 2 | Herbivorous | Macropterous | Spring |
| *Amara aulica,*  (Panzer, 1796) | Amaaul | 13 | 3 | Herbivorous | Macropterous | Autumn |
| *Amara communis,*  (Panzer, 1797) | Amacom | 7 | 2 | Herbivorous | Macropterous | Spring |
| *Amara eurynota,*  (Panzer, 1796) | Amaeur | 11 | 3 | Herbivorous | Macropterous | Spring |
| *Amara lunicollis,*  (Schiødte, 1837) | Amalun | 8 | 2 | Herbivorous | Macropterous | Spring |
| *Amara plebeja,*  (Gyllenhal, 1810) | Amaple | 7 | 2 | Herbivorous | Macropterous | Spring |
| *Amara similata,*  (Gyllenhal, 1810) | Amasim | 9 | 2 | Herbivorous | Macropterous | Spring |
| *Anchomenus dorsalis, (*Pontoppidan, 1763) | Ancdor | 7 | 2 | Generalist predator | Macropterous | Spring |
| *Anisodactylus binotatus, (*Fabricius,1787) | Anibin | 11 | 3 | Generalist predator | Macropterous | Spring |
| *Asaphidion flavipes, (*Linnaeus, 1760) | Asafla | 4 | 1 | Specialist predator | Macropterous | Spring |
| *Badister bullatus, (*Fabricius 1792) | Badbul | 5 | 2 | Generalist predator | Macropterous | Spring |
| *Bembidion lampros, (*Herbst, 1784) | Bemlam | 4 | 1 | Generalist predator | Dipolymorphic | Spring |
| *Bembidion properans,*  (Stephens, 1828) | Bempro | 4 | 1 | Generalist predator | Dipolymorphic | Spring |
| *Bembidion quadrimaculatum,* (Linnaeus, 1760) | Bemqua | 4 | 1 | Generalist predator | Macropterous | Spring |
| *Bembidion tetracolum,*  (Say, 1823) | Bemtet | 6 | 2 | Generalist predator | Dipolymorphic | Spring |
| *Calathus fuscipes,*  (Goeze, 1777) | Calfus | 12 | 3 | Generalist predator | Dipolymorphic | Autumn |
| *Calathus melanocephalus,* (Linnaeus, 1758) | Calmel | 8 | 2 | Generalist predator | Dipolymorphic | Autumn |
| *Cicindela campestris,* (Linnaeus, 1758) | Ciccam | 13 | 3 | Generalist predator | Macropterous | Spring |
| *Clivina fossor,*  (Linnaeus, 1758) | Clifos | 6 | 2 | Generalist predator | Dipolymorphic | Spring |
| *Harpalus affinis,*  (Schrank, 1781) | Haraff | 11 | 3 | Herbivorous | Macropterous | Spring |
| *Harpalus latus,*  (Linnaeus, 1758) | Harlat | 10 | 3 | Herbivorous | Macropterous | Autumn |
| *Harpalus tardus,*  (Panzer, 1797) | Hartar | 10 | 3 | Herbivorous | Macropterous | Spring |
| *Leistus ferrugineus,* (Linnaeus, 1758) | Leifer | 6 | 2 | Specialist predator | Macropterous | Autumn |
| *Leistus fulvibarbis,*  (Dejean, 1826) | Leiful | 7 | 2 | Specialist predator | Macropterous | Autumn |
| *Loricera pilicornis,* (Fabricius, 1775) | Lorpil | 7 | 2 | Specialist predator | Macropterous | Spring |
| *Nebria brevicollis,* (Fabricius, 1792) | Nebbre | 12 | 3 | Generalist predator | Macropterous | Autumn |
| *Nebria salina,*  (Fairmaire & Laboulbène, 1854) | Nebsal | 11 | 3 | Generalist predator | Macropterous | Autumn |
| *Notiophilus biggutatus,* (Fabricius, 1779) | Notbig | 5 | 2 | Specialist predator | Dipolymorphic | Spring |
| *Notiophilus quadripunctatus,*  (Dejean, 1826) | Notqua | 4 | 1 | Specialist predator | Dipolymorphic | Spring |
| *Notiophilus substriatus,*  *(*G.R. Waterhouse, 1833) | Notsub | 5 | 2 | Specialist predator | Macropterous | Spring |
| *Poecilus cupreus,* (Linnaeus, 1758) | Poecup | 11 | 3 | Generalist predator | Macropterous | Spring |
| *Poecilus versicolor,*  (Sturm, 1824) | Poever | 10 | 3 | Generalist predator | Macropterous | Spring |
| *Pseudoophonus rufipes,*  (De Geer, 1774) | Pseruf | 14 | 3 | Omnivorous | Macropterous | Autumn |
| *Pterostichus madidus,*  (Fabricius, 1775) | Ptemad | 16 | 4 | Omnivorous | Brachypterous | Autumn |
| *Pterostichus melanarius,*  (Illiger, 1798) | Ptemel | 15 | 3 | Generalist predator | Dipolymorphic | Autumn |
| *Pterostichus niger,*  (Schaller, 1783) | Ptenig | 18 | 4 | Generalist predator | Macropterous | Autumn |
| *Pterostichus strenuus,*  (Panzer, 1797) | Ptestr | 6 | 2 | Generalist predator | Dipolymorphic | Spring |
| *Pterostichus vernalis,*  (Panzer, 1796) | Ptever | 7 | 2 | Generalist predator | Dipolymorphic | Spring |
| *Stomis pumicatus,*  (Panzer, 1795) | Stopum | 7 | 2 | Generalist predator | Brachypterous | Spring |
| *Trechus quadristriatus,* (Schrank, 1781) | Trequa | 4 | 1 | Generalist predator | Macropterous | Autumn |
