# Supplementary material for: The influence of ecological infrastructures adjacent to crops on their carabid assemblages in intensive agroecosystems
Source: PeerJ. 2020 Jan 10;8:e8094. doi: 10.7717/peerj.8094 (PMC6956773; doi:10.7717/peerj.8094)
Supplement: Table S3 — Values of tested variables describing carabid assemblages in crops (activity-density, species richness, FDis index and proportion of occurence of each trait per transect) are given for each study site. [file peerj-08-8094-s003.doc]

|  | | Site n° 1 | Site n°2 | Site n°3 |
| --- | --- | --- | --- | --- |
| Activity-density | |  |  |  |
| H | C2 | 290.67 | 207 | 101.33 |
|  | C30 | 258.67 | 217 | 112 |
| G | C2 | 705 | 166 | 272 |
|  | C30 | 894 | 173 | 241 |
| H + A | C2 | 197 | 321.33 | 173 |
|  | C30 | 252 | 285.33 | 184 |
| G + A | C2 | 202 | 241.33 | 82 |
|  | C30 | 319 | 218.67 | 74 |
| Species richness | |  |  |  |
| H | C2 | 13 | 16 | 15 |
|  | C30 | 17 | 15 | 19 |
| G | C2 | 17 | 18 | 19 |
|  | C30 | 18 | 16 | 19 |
| H + A | C2 | 16 | 20 | 20 |
|  | C30 | 16 | 15 | 16 |
| G + A | C2 | 14 | 13 | 12 |
|  | C30 | 13 | 12 | 18 |
| FDis | |  |  |  |
| H | C2 | 0.236 | 0.259 | 0.250 |
|  | C30 | 0.266 | 0.253 | 0.238 |
| G | C2 | 0.263 | 0.295 | 0.230 |
|  | C30 | 0.231 | 0.294 | 0.284 |
| H+A | C2 | 0.266 | 0.318 | 0.246 |
|  | C30 | 0.288 | 0.263 | 0.275 |
| G+A | C2 | 0.192 | 0.237 | 0.337 |
|  | C30 | 0.184 | 0.291 | 0.342 |
| CWM size | |  |  |  |
| H | C2 | 6.450 | 6.947 | 6.658 |
|  | C30 | 7.639 | 7.691 | 6.976 |
| G | C2 | 10.011 | 8.151 | 7.765 |
|  | C30 | 11.775 | 8.416 | 8.552 |
| H+A | C2 | 8.643 | 10.033 | 8.538 |
|  | C30 | 10.147 | 12.093 | 10.033 |
| G+A | C2 | 10.866 | 7.304 | 12.780 |
|  | C30 | 11.561 | 8.451 | 7.297 |
| Spring breeders | |  |  |  |
| H | C2 | 0.674 | 0.725 | 0.829 |
|  | C30 | 0.624 | 0.641 | 0.857 |
| G | C2 | 0.515 | 0.530 | 0.746 |
|  | C30 | 0.319 | 0.422 | 0.610 |
| H+A | C2 | 0.641 | 0.573 | 0.613 |
|  | C30 | 0.425 | 0.322 | 0.386 |
| G+A | C2 | 0.198 | 0.773 | 0.171 |
|  | C30 | 0.150 | 0.567 | 0.703 |
| Generalist predators | |  |  |  |
| H | C2 | 0.991 | 0.908 | 0.816 |
|  | C30 | 0.943 | 0.899 | 0.786 |
| G | C2 | 0.945 | 0.783 | 0.908 |
|  | C30 | 0.979 | 0.827 | 0.880 |
| H+A | C2 | 0.843 | 0.776 | 0.844 |
|  | C30 | 0.889 | 0.888 | 0.913 |
| G+A | C2 | 0.871 | 0.862 | 0.280 |
|  | C30 | 0.912 | 0.909 | 0.716 |
| Specialist predators | |  |  |  |
| H | C2 | 0.005 | 0.087 | 0.092 |
|  | C30 | 0.036 | 0.092 | 0.143 |
| G | C2 | 0.044 | 0.193 | 0.066 |
|  | C30 | 0.010 | 0.162 | 0.108 |
| H+A | C2 | 0.059 | 0.083 | 0.104 |
|  | C30 | 0.095 | 0.047 | 0.076 |
| G+A | C2 | 0.074 | 0.122 | 0.024 |
|  | C30 | 0.0502 | 0.091 | 0.068 |
| Omnivorous | |  |  |  |
| H | C2 | 0.005 | 0.005 | 0.092 |
|  | C30 | 0.020 | 0.009 | 0.071 |
| G | C2 | 0.011 | 0.024 | 0.026 |
|  | C30 | 0.011 | 0.012 | 0.012 |
| H+A | C2 | 0.0981 | 0.141 | 0.052 |
|  | C30 | 0.016 | 0.065 | 0.011 |
| G+A | C2 | 0.054 | 0.017 | 0.6951 |
|  | C30 | 0.038 | 0.00 | 0.216 |
| Size class 1 | |  |  |  |
| H | C2 | 0.422 | 0.459 | 0.487 |
|  | C30 | 0.304 | 0.304 | 0.345 |
| G | C2 | 0.123 | 0.277 | 0.195 |
|  | C30 | 0.085 | 0.312 | 0.224 |
| H+A | C2 | 0.203 | 0.108 | 0.139 |
|  | C30 | 0.198 | 0.093 | 0.147 |
| G+A | C2 | 0.084 | 0.221 | 0.183 |
|  | C30 | 0.053 | 0.335 | 0.527 |
| Size class 2 | |  |  |  |
| H | C2 | 0.394 | 0.304 | 0.329 |
|  | C30 | 0.371 | 0.369 | 0.500 |
| G | C2 | 0.393 | 0.380 | 0.540 |
|  | C30 | 0.237 | 0.277 | 0.398 |
| H+A | C2 | 0.487 | 0.411 | 0.474 |
|  | C30 | 0.298 | 0.178 | 0.255 |
| G+A | C2 | 0.153 | 0.580 | 0.037 |
|  | C30 | 0.125 | 0.299 | 0.176 |
| Size class 3 | |  |  |  |
| H | C2 | 0.183 | 0.227 | 0.145 |
|  | C30 | 0.325 | 0.323 | 0.143 |
| G | C2 | 0.477 | 0.319 | 0.261 |
|  | C30 | 0.669 | 0.405 | 0.373 |
| H+A | C2 | 0.195 | 0.423 | 0.382 |
|  | C30 | 0.341 | 0.678 | 0.592 |
| G+A | C2 | 0.748 | 0.199 | 0.244 |
|  | C30 | 0.818 | 0.354 | 0.257 |
| Size class 4 | |  |  |  |
| H | C2 | 0 | 0.010 | 0.039 |
|  | C30 | 0 | 0.005 | 0.012 |
| G | C2 | 0.007 | 0.024 | 0.004 |
|  | C30 | 0.009 | 0.006 | 0.004 |
| H+A | C2 | 0.115 | 0.058 | 0.006 |
|  | C30 | 0.163 | 0.051 | 0.005 |
| G+A | C2 | 0.015 | 0 | 0.537 |
|  | C30 | 0.003 | 0.012 | 0.041 |
