# Supplementary figures and images for: The influence of ecological infrastructures adjacent to crops on their carabid assemblages in intensive agroecosystems
Source: PeerJ. 2020 Jan 10;8:e8094. doi: 10.7717/peerj.8094 (PMC6956773; doi:10.7717/peerj.8094)

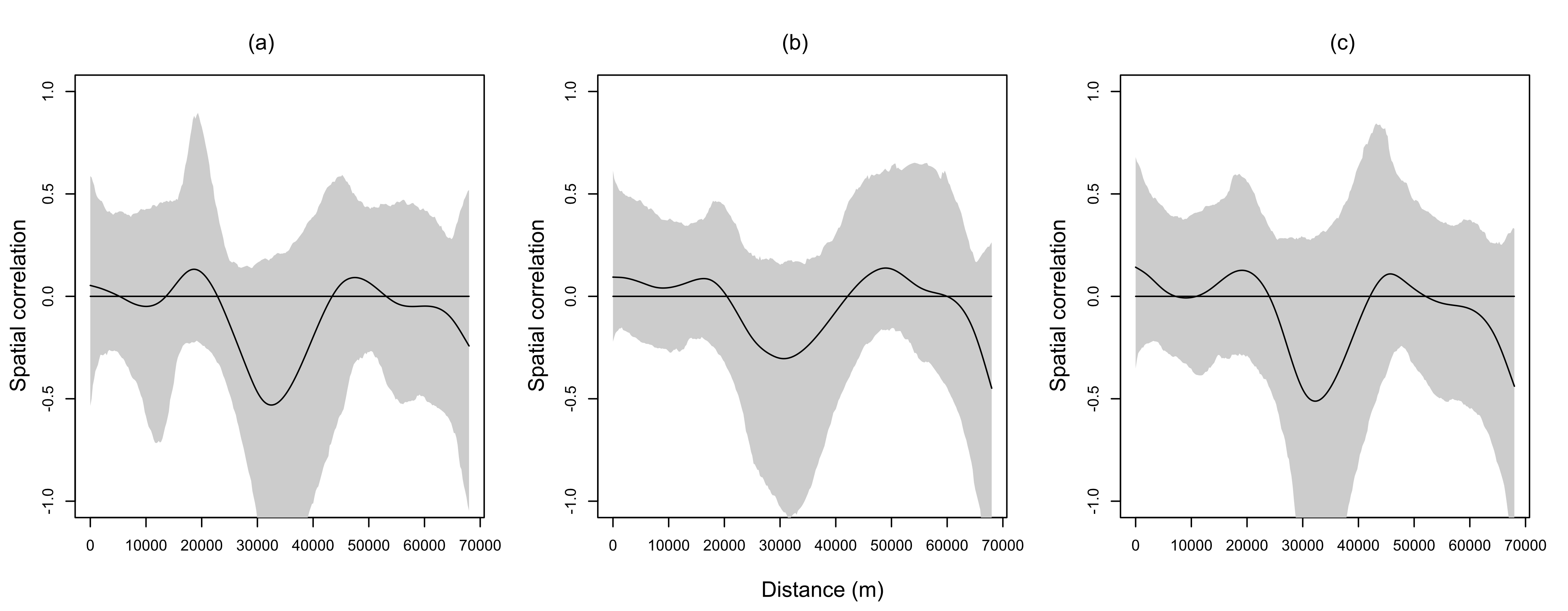

Supplement: Figure S1 — Grey zones correspond to 95% pointwise bootstrap confidence intervals. [file peerj-08-8094-s004.png]
