# Supplementary material for: The influence of ecological infrastructures adjacent to crops on their carabid assemblages in intensive agroecosystems
Source: PeerJ. 2020 Jan 10;8:e8094. doi: 10.7717/peerj.8094 (PMC6956773; doi:10.7717/peerj.8094)
Supplement: Code S1 — The following script presents the code used for the Principal Coordinate Analysis (PCoA) and the GLMM calculation. [file peerj-08-8094-s006.doc]

**R script for : “Do ecological infrastructures adjacent to crops influence their carabid assemblages in intensive agroecosystems?”**

**### Loaded libraries**

library(vegan)

library(FD)

library(ade4)

library(lme4)

library(multcomp)

library(MumIn)

library(car)

library(ncf)

**###Calculating FDis and diversity information**

car_traits=read.table(file.choose(),h=T,sep="\t",row.names="Species",na.strings="-999")

car.ab=read.table(file.choose(),h=T,sep="\t",row.names="Plot",na.strings="-999")

#Creating a list with the two tables

liste.car<-list(car.ab,car_traits)

names(liste.car) <- c("abondance", "traits")

liste.car

#Index calculation

f_index.cail<-dbFD(liste.car$traits,liste.car$abondance,corr = "cailliez")

# ANOSIM and multivariate dispersions

# Loading files

ano.tra=read.table(file.choose(),header=TRUE,row.names="Code")

ano.info=read.table(file.choose(),h=T,sep="\t",row.names="Code",na.strings="-999")

ano.dist <- vegdist(ano.tra,"bray")

attach(ano.info)

crb.ano <- anosim(Crb.dist, Paysage)

crb.ano

summary(crb.ano)

plot(crb.ano)

# Group creation

Crb.dist <- vegdist(ano.tra,"bray")

groups <- factor(c(rep(1,8), rep(2,8),rep(3,8)), labels = c("Landscape1","Landscape2","Landscape3"))

groups

## Calculate multivariate dispersions

mod <- betadisper(Crb.dist, groups)

mod

## Perform test

anova(mod)

## Permutation test for F

permutest(mod, pairwise = TRUE, permutations = 999)

**###GLMs**

car.PJtr=read.table(file.choose(),header=TRUE)

str(car.PJtr)

# Qqplots

# Activity-density

qqp(car.PJtr$Activity_density, "norm")

qqp(car.PJtr$AbondanceActivity_density , "lnorm")

gamma <- fitdistr(car.PJtr$Activity_density, "gamma")

qqp(car.PJtr$Activity_density, "gamma", shape = gamma$estimate[[1]], rate = gamma$estimate[[2]])

# Abundance

qqp(car.PJtr$Abundance, "norm")

qqp(car.PJtr$Abundance, "lnorm")

gamma <- fitdistr(car.PJtr$Abundance, "gamma")

qqp(car.PJtr$Abundance, "gamma", shape = gamma$estimate[[1]], rate = gamma$estimate[[2]])

# Richness

qqp(car.PJtrap$Richness, "norm")

qqp(car.PJtr$Richness, "norm")

qqp(car.PJtr$ Richness "lnorm")

poisson <- fitdistr(car.PJtr$Richness, "Poisson")

qqp(car.PJtr$Richness, "pois", poisson$estimate)

# FDis

qqp(car.PJtr$FD, "norm")

qqp(car.PJtr$FD, "lnorm")

gamma <- fitdistr(car.PJtr$FD, "gamma")

qqp(car.PJtr$FD, "gamma", shape = gamma$estimate[[1]], rate = gamma$estimate[[2]])

# CWM_size

qqp(car.PJtr$CWM_size, "norm")

qqp(car.PJtr$CWM_size, "lnorm")

gamma <- fitdistr(car.PJtr$CWM_size, "gamma")

qqp(car.PJtr$CWM_size, "gamma", shape = gamma$estimate[[1]], rate = gamma$estimate[[2]])

# GEN

qqp(car.PJtr$GEN, "norm")

qqp(car.PJtr$GEN, "lnorm")

gamma <- fitdistr(car.PJtr$GEN, "gamma",lower = c(0, 0))

qqp(car.PJtr$GEN, "gamma", shape = gamma$estimate[[1]], rate = gamma$estimate[[2]])

# Specialists

qqp(car.PJtr$SPE, "norm")

qqp(car.PJtr$SPE, "lnorm")

gamma <- fitdistr(car.PJtr$SPE, "gamma",lower = c(0, 0))

qqp(car.PJtr$SPE, "gamma", shape = gamma$estimate[[1]], rate = gamma$estimate[[2]])

# OH

qqp(car.PJtr$OH, "norm")

qqp(car.PJtr$OH, "lnorm")

gamma <- fitdistr(car.PJtr$OH, "gamma",lower = c(0, 0))

qqp(car.PJtr$SPE, "gamma", shape = gamma$estimate[[1]], rate = gamma$estimate[[2]])

# Spring

qqp(car.PJtr$Spring, "norm")

qqp(car.PJtr$Spring, "lnorm")

gamma <- fitdistr(car.PJtr$Spring, "gamma",lower = c(0, 0))

qqp(car.PJtr$Spring, "gamma", shape = gamma$estimate[[1]], rate = gamma$estimate[[2]])

# Fall

qqp(car.PJtrap$Fall, "norm")

qqp(car.PJtrap$Fall, "lnorm")

gamma <- fitdistr(car.PJtrap$Fall, "gamma",lower = c(0, 0))

qqp(car.PJtrap$Fall, "gamma", shape = gamma$estimate[[1]], rate = gamma$estimate[[2]])

# Class 1

qqp(car.PJtr$cl.1, "norm")

qqp(car.PJtr$cl.1, "lnorm")

gamma <- fitdistr(car.PJtr$cl.1, "gamma",lower = c(0, 0))

qqp(car.PJtr$cl.1, "gamma", shape = gamma$estimate[[1]], rate = gamma$estimate[[2]])

# Class 2

qqp(car.PJtr$cl.2, "norm")

qqp(car.PJtr$cl.2, "lnorm")

gamma <- fitdistr(car.PJtr$cl.2, "gamma",lower = c(0, 0))

qqp(car.PJtr$cl.2, "gamma", shape = gamma$estimate[[1]], rate = gamma$estimate[[2]])

# Class 3

qqp(car.PJtr$cl.3, "norm")

qqp(car.PJtr$cl.3, "lnorm")

gamma <- fitdistr(car.PJtr$cl.3, "gamma",lower = c(0, 0))

qqp(car.PJtr$cl.3, "gamma", shape = gamma$estimate[[1]], rate = gamma$estimate[[2]])

# Class 4

qqp(car.PJtr$cl.4, "norm")

qqp(car.PJtr$cl.4, "lnorm")

gamma <- fitdistr(car.PJtr$cl.4, "gamma",lower = c(0, 0))

qqp(car.PJtr$cl.4, "gamma", shape = gamma$estimate[[1]], rate = gamma$estimate[[2]])

# GLM call

# GLM

# Abundance

car.PJtr$Ab.log=log(car.PJtr$Abondance+1)

Ab1<-glm(Ab.log~ Type*Distance,data=car.PJtr,family=gaussian (link=("identity")))

Ab2<-glm(Ab.log~ Type+Distance,data=car.PJtr,family=gaussian (link=("identity")))

Ab3<-glm(Ab.log~ Distance,data=car.PJtr,family=gaussian (link=("identity")))

Ab4<-glm(Ab.log~ Type,data=car.PJtr,family=gaussian (link=("identity")))

Ab5<-glm(Ab.log~1,data=car.PJtr,family=gaussian (link=("identity")))

msAICcAb <- model.sel(Ab1, Ab2, Ab3,Ab4,Ab5)

msAICcAb

# Richness

Ric1<-glm(Richesse~ Type*Distance,data=car.PJtr,family=poisson(link=("log")))

Ric2<-glm(Richesse~ Type+Distance,data=car.PJtr,family=poisson(link=("log")))

Ric3<-glm(Richesse~ Distance,data=car.PJtr,family=poisson(link=("log")))

Ric4<-glm(Richesse~ Type,data=car.PJtr,family=poisson(link=("log")))

Ric5<-glm(Richesse~ 1,data=car.PJtr,family=poisson(link=("log")))

msAICcRic <- model.sel(Ric1, Ric2, Ric3,Ric4, Ric5)

msAICcRic

# FDis

FD1<-glm(FD~ Type*Distance,data=car.PJtr,family=gaussian (link=("identity")))

FD2<-glm(FD~ Type+Distance,data=car.PJtr,family=gaussian (link=("identity")))

FD3<-glm(FD~ Distance,data=car.PJtr,family=gaussian (link=("identity")))

FD4<-glm(FD~ Type,data=car.PJtr,family=gaussian (link=("identity")))

FD5<-glm(FD~1,data=car.PJtr,family=gaussian (link=("identity")))

msAICcFD <- model.sel(FD1,FD2,FD3, FD4, FD5)

msAICcFD

# CWM_size

CWM1<-glm(CWM_size~ Type*Distance,data=car.PJtr,family=gaussian (link=("identity")))

CWM2<-glm(CWM_size~ Type+Distance,data=car.PJtr,family=gaussian (link=("identity")))

CWM3<-glm(CWM_size~ Distance,data=car.PJtr,family=gaussian (link=("identity")))

CWM4<-glm(CWM_size~ Type,data=car.PJtr,family=gaussian (link=("identity")))

CWM5<-glm(CWM_size~1,data=car.PJtr,family=gaussian (link=("identity")))

msAICcCWM <- model.sel(CWM1,CWM2,CWM3,CWM4,CWM5)

msAICcCWM

Anova(CWM4)

summary(glht(CWM4, mcp(Type="Tukey")))

# Spring/Autumn breeders

Spg1<-glm(Spring~ Type*Distance,data=car.PJtr,family=gaussian (link=("identity")))

Spg2<-glm(Spring~ Type+Distance,data=car.PJtr,family=gaussian (link=("identity")))

Spg3<-glm(Spring~ Distance,data=car.PJtr,family=gaussian (link=("identity")))

Spg4<-glm(Spring~ Type,data=car.PJtr,family=gaussian (link=("identity")))

Spg5<-glm(Spring~1,data=car.PJtr,family=gaussian (link=("identity")))

msAICcSpg <- model.sel(Spg1,Spg2,Spg3,Spg4, Spg5)

msAICcSpg

Anova(Spg4)

Anova(Spg3)

summary(glht(Spg4, mcp(Type="Tukey")))

# GEN

GEN1<-glm(GEN~ Type*Distance,data=car.PJtr,family=gaussian (link=("identity")))

GEN2<-glm(GEN~ Type+Distance,data=car.PJtr,family=gaussian (link=("identity")))

GEN3<-glm(GEN~ Distance,data=car.PJtr,family=gaussian (link=("identity")))

GEN4<-glm(GEN~ Type,data=car.PJtr,family=gaussian (link=("identity")))

GEN5<-glm(GEN~1,data=car.PJtr,family=gaussian (link=("identity")))

msAICcGEN <- model.sel(GEN1,GEN2,GEN3,GEN4,GEN5)

msAICcGEN

Anova(GEN3)

# SPE

SPE1<-glm(SPE~ Type*Distance,data=car.PJtr,family=gaussian (link=("identity")))

SPE2<-glm(SPE~ Type+Distance,data=car.PJtr,family=gaussian (link=("identity")))

SPE3<-glm(SPE~ Distance,data=car.PJtr,family=gaussian (link=("identity")))

SPE4<-glm(SPE~ Type,data=car.PJtr,family=gaussian (link=("identity")))

SPE5<-glm(SPE~1,data=car.PJtr,family=gaussian (link=("identity")))

msAICcSPE <- model.sel(SPE1,SPE2,SPE3,SPE4,SPE5)

msAICcSPE

# OH

car.PJtr$OH.log=log(car.PJtr$OH+1)

OH1<-glm(OH.log~ Type*Distance,data=car.PJtr,family=gaussian (link=("identity")))

OH2<-glm(OH.log~ Type+Distance,data=car.PJtr,family=gaussian (link=("identity")))

OH3<-glm(OH.log~ Distance,data=car.PJtr,family=gaussian (link=("identity")))

OH4<-glm(OH.log~ Type,data=car.PJtr,family=gaussian (link=("identity")))

OH5<-glm(OH.log~1,data=car.PJtr,family=gaussian (link=("identity")))

msAICcOH <- model.sel(OH1,OH2,OH3,OH4,OH5)

msAICcOH

Anova(OH3)

# Class 1

Cl11<-glm(cl.1~ Type*Distance,data=car.PJtr,family=gaussian (link=("identity")))

Cl12<-glm(cl.1~ Type+Distance,data=car.PJtr,family=gaussian (link=("identity")))

Cl13<-glm(cl.1~ Distance,data=car.PJtr,family=gaussian (link=("identity")))

Cl14<-glm(cl.1~ Type,data=car.PJtr,family=gaussian (link=("identity")))

Cl15<-glm(cl.1~1,data=car.PJtr,family=gaussian (link=("identity")))

msAICcCl1 <- model.sel(Cl11,Cl12,Cl13,Cl14, Cl15)

msAICcCl1

Anova(Cl14)

summary(glht(Cl14, mcp(Type="Tukey")))

# cl2

Cl21<-glm(cl.2~ Type*Distance,data=car.PJtr,family=gaussian (link=("identity")))

Cl22<-glm(cl.2~ Type+Distance,data=car.PJtr,family=gaussian (link=("identity")))

Cl23<-glm(cl.2~ Distance,data=car.PJtr,family=gaussian (link=("identity")))

Cl24<-glm(cl.2~ Type,data=car.PJtr,family=gaussian (link=("identity")))

Cl25<-glm(cl.2~1,data=car.PJtr,family=gaussian (link=("identity")))

msAICcCl2 <- model.sel(Cl21,Cl22,Cl23,Cl24, Cl25)

msAICcCl2

Anova(Cl23)

# cl3

car.PJtr$cl3.log=log(car.PJtr$cl.3+1)

Cl31<-glm(cl.3~ Type*Distance,data=car.PJtr,family=gaussian (link=("identity")))

Cl32<-glm(cl.3~ Type+Distance,data=car.PJtr,family=gaussian (link=("identity")))

Cl33<-glm(cl.3~ Distance,data=car.PJtr,family=gaussian (link=("identity")))

Cl34<-glm(cl.3~ Type,data=car.PJtr,family=gaussian (link=("identity")))

Cl35<-glm(cl.3~1,data=car.PJtr,family=gaussian (link=("identity")))

msAICcCl3 <- model.sel(Cl31,Cl32,Cl33,Cl34, Cl35)

msAICcCl3

Anova(Cl33)

# cl4

car.PJtr$cl4.log=log(car.PJtr$cl.4+1)

Cl41<-glm(cl.4~ Type*Distance,data=car.PJtr,family=gaussian (link=("identity")))

Cl42<-glm(cl.4~ Type+Distance,data=car.PJtr,family=gaussian (link=("identity")))

Cl43<-glm(cl.4~ Distance,data=car.PJtr,family=gaussian (link=("identity")))

Cl44<-glm(cl.4~ Type,data=car.PJtr,family=gaussian (link=("identity")))

Cl45<-glm(cl.4~1,data=car.PJtr,family=gaussian (link=("identity")))

msAICcCl4 <- model.sel(Cl41,Cl42,Cl43,Cl44,Cl45)

msAICcCl4

Anova(Cl43)

**#### Checking for spatial auto-correlation with spline-correlograms and GLS models**

library(ncf)

# CWM_size

CWM4<-glm(CWM_size~ Type,data=car.PJtr,family=gaussian (link=("identity")))

corr.CWM<-spline.correlog(x =car.PJtr[, "Long"],y = car.PJtr[, "Lat"],z = residuals(CWM4, type =

"pearson") )

summary(corr.CWM)

plot(corr.CWM)

# Spring/Autumn breeders

Spg4<-glm(Spring~ Type,data=car.PJtr,family=gaussian (link=("identity")))

corr.Spg4<-spline.correlog(x =car.PJtr[, "Long"],y = car.PJtr[, "Lat"],z = residuals(Spg3, type =

"pearson") )

summary(corr.Spg4)

plot(corr.Spg4)

# Class 1

Cl14<-glm(cl.1~ Type,data=car.PJtr,family=gaussian (link=("identity")))

corr.Cl1<-spline.correlog(x =car.PJtr[, "Long"],y = car.PJtr[, "Lat"],z = residuals(Cl14, type =

"pearson") )

summary(corr.Cl1)

plot(corr.Cl1)

### GLS corrected

gls.c1<-gls(cl.1~ Type,car.PJauto)

gls.spg<-gls(Spring~ Type,car.PJauto)

gls.CWM<-gls(CWM_size~ Type,car.PJauto)

# Cl1

M4a <- update(gls.c1, correlation = corExp(form=~ "Long" + "Lat", nugget = TRUE))

M4b <- update(gls.c1, correlation = corLin(form=~ "Long" + "Lat", nugget = TRUE))

M4c <- update(gls.c1, correlation = corGaus(form=~ "Long" + "Lat", nugget = TRUE))

M4d <- update(gls.c1, correlation = corSpher(form=~ "Long" + "Lat", nugget = TRUE))

M4e <- update(gls.c1, correlation = corRatio(form=~ "Long" + "Lat", nugget = TRUE))

AIC(gls.c1, M4a, M4b, M4c, M4d, M4e)

# Spring breeders

M5a <- update(gls.spg, correlation = corExp(form=~ "Long" + "Lat", nugget = TRUE))

M5b <- update(gls.spg, correlation = corLin(form=~ "Long" + "Lat", nugget = TRUE))

M5c <- update(gls.spg, correlation = corGaus(form=~ "Long" + "Lat", nugget = TRUE))

M5d <- update(gls.spg, correlation = corSpher(form=~ "Long" + "Lat", nugget = TRUE))

M5e <- update(gls.spg, correlation = corRatio(form=~ "Long" + "Lat", nugget = TRUE))

AIC(gls.spg, M5a, M5b, M5c, M5d, M5e)

# CWM

M6a <- update(gls.CWM, correlation = corExp(form=~ "Long" + "Lat", nugget = TRUE))

M6b <- update(gls.CWM, correlation = corLin(form=~ "Long" + "Lat", nugget = TRUE))

M6c <- update(gls.CWM, correlation = corGaus(form=~ "Long" + "Lat", nugget = TRUE))

M6d <- update(gls.CWM, correlation = corSpher(form=~ "Long" + "Lat", nugget = TRUE))

M6e <- update(gls.CWM, correlation = corRatio(form=~ "Long" + "Lat", nugget = TRUE))

AIC(gls.CWM, M6a, M6b, M6c, M6d, M6e)

**###PCoA Carabids**

library(vegan)

Crb.acop=read.table(file.choose(),header=TRUE,row.name="Plot")

d14=sqrt(vegdist (Crb.acop, method = "bray"))

pcoa <- cmdscale(d14, add= TRUE, eig= TRUE)

pcoa

pcoa1 = pcoa$points[,1]

pcoa2 = pcoa$points[,2]

coordo = data.frame(pcoa1, pcoa2, row.names=rownames(Crb.acop))

eigen_values = round(pcoa$eig/sum(pcoa$eig)*100, digits = 2)

barplot(eigen_values, col = "chocolate")

eigen_values

fig.sites <- ordiplot(pcoa, type="none")

abline(h=0, lty=3); abline(v=0, lty=3)

points(pcoa1, pcoa2,cex=0.8,pch=20, col = "black")

**###Adding centroids of EI and crops**

# Creating factors for ordispider

grp.acop= substr(rownames(Crb.acop),3,3)

grp.acop

ordispider(pcoa, factor(grp.acop), label = TRUE)

#Adding carabid species

caraba=read.table(file.choose(),header=TRUE,row.name="Species") # table with species list and their global abundance

caraba$ab.log=log((caraba$Abondance)+1)

pcoa.wa <- wascores(pcoa$points[,1:2],Crb.acop)

fig.sp <- ordiplot(pcoa, type="none")

abline(h=0, lty=3); abline(v=0, lty=3)

# simple points

points(pcoa.wa,col="black",pch = 16,cex=0.7)

text(pcoa.wa, rownames (pcoa.wa), cex=0.7, col ="black")

# with a circle of size proportionnal to species abundance

points(pcoa.wa,col = "gray35",cex=(caraba$ab.log))
